# Enlarged Prostate—Suppressed Urine—Remedy

**Published:** 1872-08

**Authors:** J. C. Kurt

**Affiliations:** Talladega, Ala.


### ENLARGED PROSTATE—SUPPRESSED URINE—REMEDY.

BY J. C. KURT, M. D., TALLADEGA, ALA.

There is perhaps no other gland in the human system, of apparently so little importance to its economy, which has given the physician and his patients so much trouble as the prostate. Situated as it is, just between the two great outlets of the system, the urethra and rectum, securely attached to the former, and partially to the latter, it is not surprising that it should be greatly affected by any active disease in either. Hence, we frequently find the gland engorged, or congested and swollen, so that it presses upon the urethra, shutting it up, and obstructing entirely the passage of urine. When this occurs, then trouble begins to the patient as well as to his physician, unless proper means of relief are resorted to at an early period of the obstruction—which is rarely called for by the patient, often waiting and hoping by the use of some simple means to get relief, until the difficulty becomes quite grave—the necessity for relief urgent, and the danger eminent.

It was under these circumstances that the writer, several years since, was called to see his first case of this kind. It was a young negro man, about eighteen years of age, apparently in good health, except, as he stated, the obstruction of his urine, which he said had then existed for twenty-four hours or more. The bladder could be distinctly felt through the walls of the abdomen, much enlarged, very tense, and exceedingly painful at short intervals, with the straining and ineffectual efforts at micturition.

After many unsuccessful efforts, with a variety of instruments, and in various positions of the patient, to pass a catheter, and having tried the warm bath and other simple means, and all having utterly failed, and now, the symptoms becoming more urgent every hour—the patient in an agony in body, and his physician in quite as much of an agony mentally and professionally—it was manifest that some relief must be had soon, or the consequences might prove serious. Believing that a remedy which would produce speedy and complete relaxation of the system, might relieve the difficulty, it was determined to try venesection, upon Marshall Hall's plan of standing the patient upright, and opening a large orifice in each arm, so as to secure the desired relaxation, with the least possible loss of blood. This was done, and to the great gratification of both physician and patient, about the time incipient syncope was manifest the urine flowed freely.

The second case which occurred to the writer, was one in which a "brother chip" had been in attendance for twenty-four hours or more, and had been completely foiled in all his efforts to relieve by catheters, and all other means that he had tried. The writer was called in consultation The patient was a youth about sixteen years old (white). Finding that all the ordinary means of relief had been perseveringly used for a considerable time, and no relief obtained, it was decided to try venesection, upon the same plan that had proved successful in my first case. This was done, and the result was prompt and complete relief.

The same remedy has been since tried by the writer in a third and fourth case—the latter only a few days since, in an old man above sixty years of age. Some hesitation was felt in trying such a remedy on such a patient, but when all other means had failed to give relief, and the patient seemed to be in such an agony that he could not live long, this was tried, and relieved him promptly.

I shall not enter into any theoretical explanations in regard to this remedy. The facts are given as they occurred. Others with more skill might have passed the catheter successfully, or found some other means of relief. I am aware that many physicians of the present time will be ready to pronounce this practice merely a relict of "old fogyism," as they fain to regard blood letting among the "obsolete remedies" of a past age. Nevertheless, it is still the means of prompt and safe relief in the above mentioned cases, and many others which might be mentioned, when all others known to the writer have signally failed.

I have had opportunity to know the history of the persons upon whom this remedy was used—some of them for several years—and there has not been any return of the difficulty. This, however, cannot be attributed to any influence of the remedy.

These cases have been furnished principally in compliance with a request made in the June number of the COMPANION, by Geo. F. Taylor, M. D., of Alabama, and hoping that they may be suggestive to the junior members of the profession in similar difficulties.

